# Robust IoT security using isolation forest and one class SVM algorithms

**DOI:** 10.1038/s41598-025-20445-4

**Published:** 2025-10-21

**Authors:** Amna Zahoor, Waseem Abbasi, Muhammad Zeeshan Babar, Abeer Aljohani

**Affiliations:** 1 Department of Computer Science, The University of Lahore, Sargodha Campus, Sargodha, 40100 Pakistan; 2 School of Engineering and Physical Sciences, Heriot Watt University, Edinburgh, EH144AS UK; 3 Department of Computer Science and Informatics, Applied College, Taibah University, 42353 Madinah, Saudi Arabia

**Keywords:** Intrusion detection systems (IDS), IoT security, Anomaly detection, Machine learning, Cyber threats, Engineering, Mathematics and computing

## Abstract

The rapid growth of cloud computing and the Internet of Things (IoT) has increased the exposure of IoT devices to cyber-attacks due to their resource limitations and lack of standardized security protocols. This paper presents a robust anomaly detection framework for IoT networks using two unsupervised machine learning models: Isolation Forest (IF) and One-Class Support Vector Machine (OCSVM). Leveraging the TON_IoT dataset, we conduct a comparative evaluation of IF, OCSVM, and a lightweight fusion approach called Combined Scoring Anomaly Detection (CSAD). Results show that OCSVM achieves superior precision, recall, and accuracy compared to both IF and CSAD. To ensure reliability, we apply Random Forest-based feature importance analysis, fivefold cross-validation and hyperparameter tuning. Model resilience is further examined under adversarial label-flip poisoning attacks and interpretability is enhanced through Local Interpretable Model-Agnostic Explanations (LIME). The findings demonstrate that lightweight unsupervised algorithms can provide effective, low-resource anomaly detection for modern IoT environments.

## Introduction

**Figure1:**
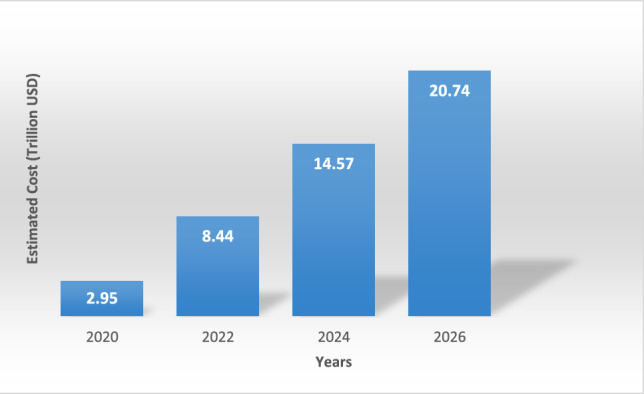
Estimated costs of future cybercrimes overtime.

The rapid growth and deployment of IoT devices has made them an essential part of modern life, driven by their affordability, availability and convenience^1^. IoT devices consist of sensors, software, processing power and similar technologies that communicate and exchange data over the Internet. IoT networks are applied in industrial, commercial, consumer and infrastructure domains, including home automation, wearables, remote health monitoring, digital control, smart cities and traffic management^2^. However, the sensitive information collected and transmitted by these devices is increasingly exposed to cyber threats, resulting in potential privacy violations. With the growing adoption of IoT, the security of devices has become a critical issue. Companies face higher costs for IoT network security, attack recovery and device maintenance. The financial impact of cyberattacks on governments over the year isillustrated in Fig. 1. IoT data is typically transmitted to cloud platforms for processing and storage, which supports continuous monitoring, remote access and large-scale analysis but also increases the risks of security breaches and privacy violations^3^.The abbreviations used throughout this paper are listed in Table 1.Given these vulnerabilities and the increasing reliance on IoT devices, it becomes essential to understand the motivations behind anomaly detection research as well as the key challenges that hinder secure deployments.

**Table 1:** List of Abbreviations.

Abbreviations	Full form
IoT	Internet of things
IDS	Intrusion detection system
IF	Isolation forest
OCSVM	one-class support vector machine
CSAD	Combined scoring anomaly detection
RF	Random forest
LIME	Local interpretable model-agnostic explanations
DoS	Denial of service
DNN	Deep neural network
CNN	Convolutional neural network
RNN	Recurrent neural network
LSTM	Long short-term memory
GRU	Gated recurrent unit
ANN	Artificial neural network
AE	Autoencoder
TL	Transfer learning
BiLSTM	Bidirectional long short-term memory
SHAP	Shapley additive explanations
CPS	Cyber-physical system
SMOTE	Synthetic minority oversampling technique
ResNet	Residual networks
CV	Cross-validation
NIZKPs	Non-interactive zero-knowledge proofs
TP	True positive
TN	True negative
FP	False positive
FN	False negative
ELM	Extreme learning machine
NIDS	Network-based intrusion detection system
IoMT	Internet of medical things
IIoT	Industrial internet of things
GA	Genetic algorithm

### Motivations and challenges

The number of IoT devices is projected to reach 26 billion by 2030, producing up to 73.1 zettabytes of data by 2025. This massive growth makes manual monitoring impractical and highlights the urgent need for automated and reliable anomaly detection systems^4^.A major challenge lies in the resource-constrained nature of IoT devices,such as limited energy and processing power,which prevents them from implementing advanced security protocols.In addition, IoT devices lack standardized development guidelines or universally accepted frameworks, making them highly vulnerable to Internet-based threats^5^. Another persistent issue is the constant emergence of new attack vectors, a direct consequence of the highly interconnected structure of IoT networks.

One potential solution for mitigating cyberattacks is the deployment of Intrusion Detection Systems (IDS). IDS technologies perform real-time monitoring to identify threats, suspicious events and security breaches. However, these solutions are not always effective. For example, Network-Based Intrusion Detection Systems (NIDS) often generate many false positives when detecting zero-day attack anomalies^6^.Moreover, traditional IDS approaches are limited because they rely on static attack signatures and are unable to detect emerging threats.As a result, anomaly detection in IoT environments has become a critical research priority that requires prompt and effective solutions^7^. Our research aims to address a critical gap in the current literature regarding the limitationsof existing IDS.Several ML techniques have been proposed to overcome these challenges. Among them, unsupervised algorithms such as IF and OCSVM have gained attention because they can detect abnormal behaviors without relying on labeled data. However, priorstudies often overlook important aspects, including the robustness of these models and their statistical validation^8^.In particular, there is limited research on how unsupervised models respond topoisoning attacks and how feature importance influences their decision-making processes. To address these gaps, this paper proposes an anomaly detection framework designed to enhance the security of IoT systems. The framework integrates IF and OCSVM models and is evaluated on the TON_IoT dataset, demonstrating effectiveness and producing reliable predictions for identifying malicious activity.

### Research contributions

The main contributions of this research are summarized as follows:

1. A detailed comparison of two unsupervised machine learning (ML) models—OCSVM and IF—on the TON_IoT dataset, highlighting their relative strengths in detecting anomalies within IoT networks.
2. Development of a CSAD approach that integrates anomaly scores from OCSVM and IF, thereby improving anomaly detection performance through score-level fusion.
3. Application of LIME to explain the decision-making process of unsupervised models, addressing the common gap of limited interpretability in prior anomaly detection studies.
4. Assessment of model reliability under adversarial scenarios, such as label-flip poisoning attacks, to evaluate the resilience of OCSVM and IF in practical IoT attack environments.
5. Incorporation of Random Forest-based feature importance ranking**,** fivefold cross-validation and hyperparameter tuning to ensure reproducibility and strengthen the statistical validity of results.

To situate these contributions within the broader research landscape, we next review recent work on anomaly detection in IoT, highlighting existing strengths and unresolved gaps.

### Paper organization

Our paper is organized as follows: "Related work" presents a review of the related literature. "Methodology" describes the methodology in detail, along with the dataset used in this study. "Implementation and result analysis" reports and discusses the findings obtained from the proposed model. Finally, "Discussion" concludes the paper and outlines possible directions for future work.

### Related work

This section reviews existing approaches for anomaly detection in IoT network traffic, emphasizing both their strengths and limitations. A summary of recent approaches is provided in Table 2.It also highlights how our proposed framework addresses these shortcomings and advances the state of the art in IoT anomaly detection.The study by Himanshu Nandanwar et al.^9^ introduces Cyber-Sentinet, a deep learning-based IDSdesigned for cyber-physical system (CPS) security. The model combines 2D Convolutional Neural Networks (CNN) and Residual Networks (ResNet) to capture spatial and temporal features. It also uses SHAP explanations to make the model’s decisions easier for security experts to understand and trust.The model achieved 97.46% accuracy on theEdge-IIoT-2022dataset, surpassing several state-of-the-art approaches. This work enhances the resilience and reliability of CPS in the context of Industry 5.0. It addresses emerging cybersecurity challenges in Industrial IoT (IIoT) environments. A recent study^10^ proposed a deep learning framework called AttackNet, developed for detecting and classifying diverse botnet attacks in IIoT environments. The architecture integrates Convolutional Neural Networks (CNN) with Gated Recurrent Units (GRU) and was evaluated using the N-BaIoT dataset. AttackNet achieved remarkable performance, with an accuracy of 99.75% and a validation loss of 0.0063, surpassing other contemporary deep learning approaches. An ablation study further analyzed the contribution of each component to the model’s effectiveness. Nandanwar et al.^11^ also developed a transfer learning model, TL-Bidirectional Long Short-Term Memory (TL-BiLSTM), for detecting botnet attacks in IoT. The framework integrates CNN with BiLSTM layers to classify attacks such as Mirai and BASHLITE across nine types of IoT devices. Using a real-world dataset composed of both benign and malicious network traffic, the model achieved testing and training accuracies of 99.52% and 99.55%, respectively. When applied to the N-BaIoT dataset, TL-BiLSTM outperformed several existing detection techniques. Further, a separate study^12^ proposed a decentralized blockchain-based application to enhance security and privacy in IoT-enabled healthcare systems. The solution employs smart contracts to enable secure interaction among patients, healthcare providers and IoT devices, effectively reducing threats such as phishing and identity theft. It alsoincorporates Non-Interactive Zero-Knowledge Proofs(NIZKPs) for data privacy, uses the Inter-Planetary File System (IPFS) for secure data storage and operates on Ethereum smart contracts. In addition, an embedded IDS monitors network traffic to detect potential threats, offering a scalable and robust framework for managing healthcare data.

**Table2:** Summary of Related Work.

Author & Year	Detection Model	Targeted environment	Accuracy	Dataset	Noted limitations
Nandanwar et al.^9^	2D-CNN, ResNet	Industry 5.0	97.46%	Edge-IIoT-2022	Not evaluated under adversarial or real-time constraints
Nandanwar et al.^10^	CNN, GRU (AttackNet)	Industrial IoT	99.75%	N_BaIoT	High compute demands; interpretability not addressed
Nandanwar et al.^11^	Hybrid CNN-BiLSTM + TL	IoT Networks	99.52%	N_BaIoT	Requires extensive labeled data for transfer learning
Nandanwar et al.^12^	Blockchain-based IDS	IoT healthcare	N/A	N/A	No quantitative performance metrics reported
Esra et al.^13^	DT, RF, kNN, SVM	IoT Networks	~ 99% +	IoTID20	Limited to accuracy; no robustness or interpretability study
Khalid et al.^14^	DT, LR, XG Boost	IoT Networks	94%	UNSW-NB15	No evaluation under poisoning or adversarial settings
Imtiaz et al.^15^	CNN1D/2D/3D	IoT Networks	99% +	BoT-IoT, MQTT-IoT-IDS2020, IoT-23	Not tested on resource-constrained hardware
Anshika et al.^16^	DT, LR, SVM, RF	IoT Networks	RF: 98.47%, SVM: 92.8%	N/A	No benchmark with deep learning or ensemble approaches
Zeeshan et al.^17^	DNN	IoT Networks	99.01%	IoT-Botnet 2020	No explainability or poisoning attack analysis
Dheyaaldin^18^	FusionNet	IoMT	98–99%	WUSTL EHMS, ICU-IoMT	No comparison with simpler unsupervised baselines
Nadeem et al.^19^	RF, ANN, DT, LSTM, AdaBoost, AE	Smart Homes	Up to 100%	UNSW BoT-IoT	Lacks interpretability and adversarial robustness analysis
Maryam et al.^20^	RF, DT, LR, Perceptron, AdaBoost	Healthcare	RF: 99.555%	CIC IoT	No discussion of runtime overhead or edge feasibility
Lerinaetal.^21^	DNN	IoT Networks	99.89%	N/A	High accuracy, but lacking in ACM real-time or interpretability evaluation
Abu Al-Haija et al.^22^	ELM – survey of variants (S-ELM, U-ELM, Semi-ELM)	Network & IoT intrusion detection (IDS)	Varies	NSL-KDD, CIC-IDS2017, BoT-IoT	Scalability issues on large datasets, potential overfitting, limited handling of multimodal inputs
Altamimi& Abu Al-Haija^23^	ELM	IoT networks (IDS)	NSL-KDD: 99.6% (bin.), 92.5% (multi); Distilled-Kitsune: 99.9% +	NSL-KDD (2009), Distilled-Kitsune (2021)	Limited handling of highly non-linear attacks; requires tuning for real-world use

A research in^13^ introduced an IDS aimed at bolstering cybersecurity in IoT environments by identifying and responding to Denial of Service (DoS) attacks. The research evaluated various ML classifiers including Decision Trees (DT), Random Forests (RF), Support Vector Machines (SVM) and K-Nearest Neighbor (kNN).Their efficiency was compared based on training and testing durations. Results showed that DT and RF performed best, especially when combined with Genetic Algorithm (GA)-based feature selection. DT also demonstrated the highest computational efficiency. Another study by Khalid Alissa and collaborators^14^ applied ML algorithms to detect and classify botnet attacks using the UNSW-NB15 dataset. To address class imbalance, the Synthetic Minority Oversampling Technique (SMOTE) was employed. Among the models evaluated—DT, Extreme Gradient Boosting XGBoost), and Logistic Regression (LR)—DT achieved the highest detection accuracy, identifying botnet activity with a 94% success rate. Additionally, a comprehensive review^15^ explored contemporary deep learning methods designed to strengthen IoT security. Researchers developed multi-class classification models based on one-, two-, and three-dimensional CNN architectures. These models were evaluated on several intrusion detection datasets, including BoT-IoT, MQTT-IoT-IDS2020, IoT Network Intrusion, and IoT-23. The study also utilized Recursive Feature Elimination (RFE) to optimize input feature selection. Anshika Sharma et al.^16^ proposed an anomaly detection solution using LR, DT, SVM and RF. Among these, RF achieved the highest accuracy (98.47%), outperforming the others. Zeeshan Ahmad et al.^17^ focused on detecting IoT networkvulnerabilities using a Deep Neural Network (DNN). Mutual Information (MI) was applied to identify the most important features. The performance of CNN, DNN and Recurrent Neural Network (RNN) variations—including Gated Recurrent Unit (GRU) and Long Short-Term Memory (LSTM)—was compared using the Botnet 2020 dataset. Results showed that the proposed DNN-based model outperformed existing deep learning methods, achieving a detection accuracy of 99.01% with a False Alarm Rate (FAR) of 3.9%.

In^18^, researchers proposed FusionNet, an ensemble model to improve IoMT security. It combines the strengths of RF, SVM, kNN and MLP. FusionNet was tested on two datasets and compared against individual models such as RF, SVM and kNN. Results showed that it achieved 98.5% accuracy on Dataset 1 and 99.5% on Dataset 2. Additionally, the study integrated blockchain to ensure access control and maintain data integrity. Nadeem Sarwar et al.^19^ highlighted the need for smart home security solutions. They proposed a technique using RF, DT, LSTM, AdaBoost and ANN for anomaly detection, evaluated on the UNSW BoT-IoT dataset. Feature selection and encoding methods were applied to balance the data. Results showed that RF, DT and AdaBoost achieved high accuracy, while ANN performed comparatively worse. In^20^, the authors reviewed the role of MIoT devices in healthcare. They developed models using RF, AdaBoost, LR, Perceptron and DNN to detect binary, multiclass and 34-class attacks. Evaluation on the CCIoT2023 dataset, which includes 33 IoT attack types, showed that RF performed best, particularly in classifying attacks across 2, 8, and 34 categories.

The work by Abu Al-Haija et al.^21^ conducted a comprehensive survey of Extreme Learning Machines (ELMs) in the context of intelligent IDSs. ELM, a variant of single-hidden-layer feedforward neural networks, has gained attention due to its fast training speed, reduced computational complexity and suitability for real-time applications. The survey reviewed over 170 studies from the past decade, categorizing them into supervised, unsupervised and semi-supervised ELM approaches. The authors highlighted ELM’s effectiveness in detecting DDoS attacks, phishing and malicious traffic while also exploring hybrid IDS designs that combine ELM with other algorithms such as GA, SVM and clustering techniques. Importantly, the paper emphasized that while ELM provides efficiency and adaptability, challenges remain in handling large datasets, multimodal data and overfitting issues. The study concluded that ELM represents a promising alternative for lightweight, fast and accurate IDS development, but further research is needed to improve scalability and robustness. In another work Altamimi and Abu Al-Haija^22^ proposed an ELM-based IDS for IoT networks. The model was evaluated on NSL-KDD (2009) and Distilled-Kitsune (2021) datasets for both binary and multi-class classification tasks. Their experiments showed that ELM consistently outperformed conventional models such as KNN, DT and RF, achieving up to 99.9% F1-score in binary classification and 99.98% in multi-class classification. The study highlighted ELM’s strengths in scalability, low computational cost and robustness against imbalanced traffic data. However, limitations remain in capturing highly non-linear attack patterns and ensuring robustness for real-world IoT deployments.

In^23^, the authors proposed a fraud detection method for Bitcoin networks by integrating AI with blockchain. The approach used XGBoost and RF to classify transactions and proved effective against Sybil and double-spending attacks. They also presented a smart contract security analysis and an attacker model to further assess system resilience.In another article^24^, the authors reviewed the core concepts of blockchain technology, emphasizing its decentralized and immutable nature, which enables secure storage of data collected from IoT devices. The study also examined the integration of ML with blockchain to enable intelligent and trustworthy data analysis.In^25^, the researchers discussed the benefits of integrating IoT technology with ML to enhance the security of smart manufacturing plants. In industrial environments, robust security measures are essential to ensure operational safety. The study also emphasized the importance of adapting security solutions to counter emerging threats. Building on the insights from prior studies, we now present the methodology adopted in this research, including dataset details, preprocessing steps and the proposed anomaly detection framework.

## Methodology

**Fig. 2:**
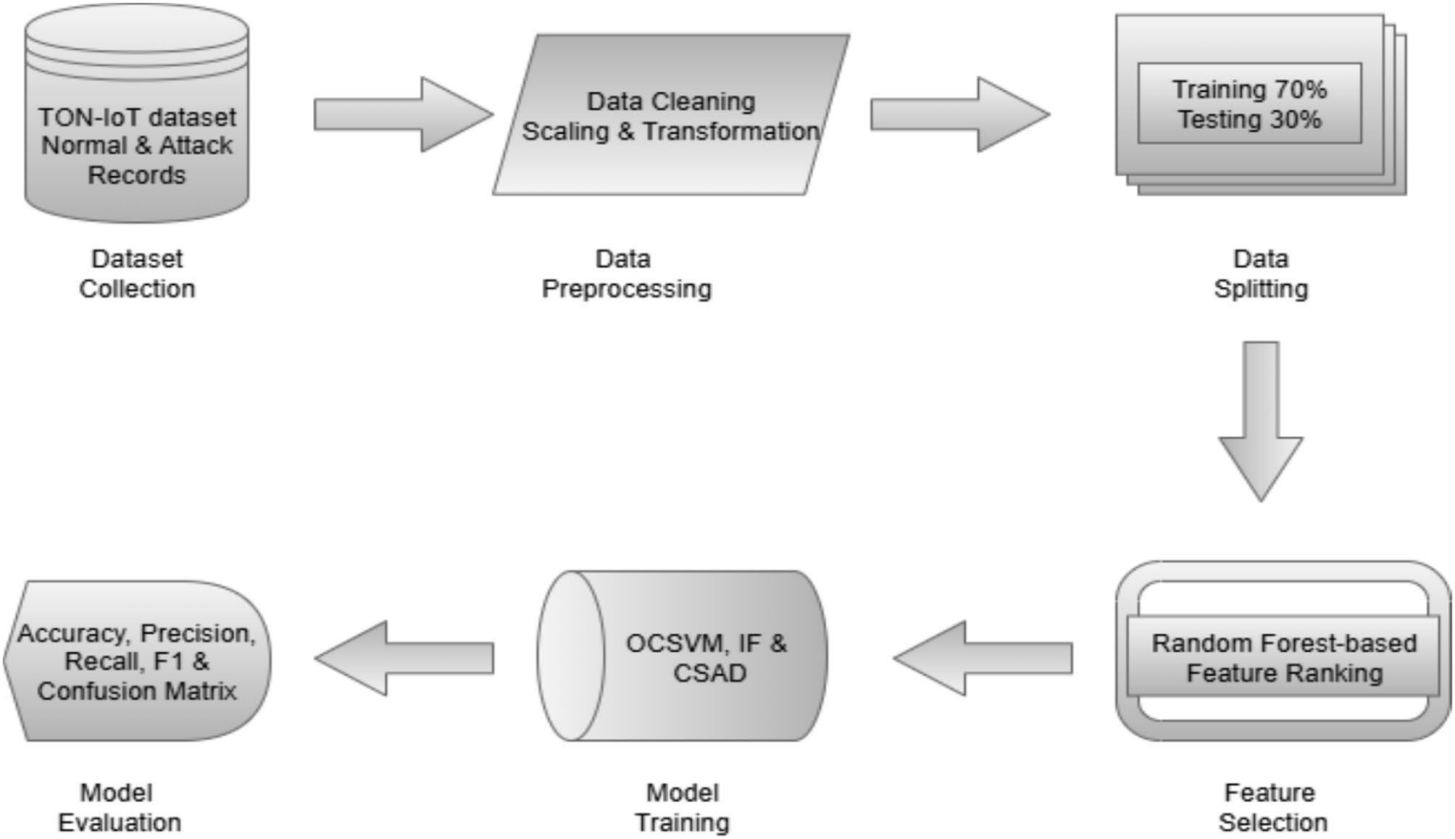
Proposed methodology for anomaly detection in IoT networks using OCSVM, IF, and CSAD.

The methodology adopted for detecting anomalies in IoT networks is outlined in this section. While IoT devices offer connectivity and convenience, data transmitted to the cloud is vulnerable to attacks. Without proper updates and security, these devices can be exploited for disruption, unauthorized access, or data theft^26^. To address these challenges, this paper proposes an anomaly detection framework using two unsupervised MLalgorithms: One-Class Support Vector Machine (OCSVM) andIsolation Forest (IF). The proposed framework is structured as an ML layered architecture, as illustrated in Fig. 2.

### Dataset collection

**Fig. 3:**
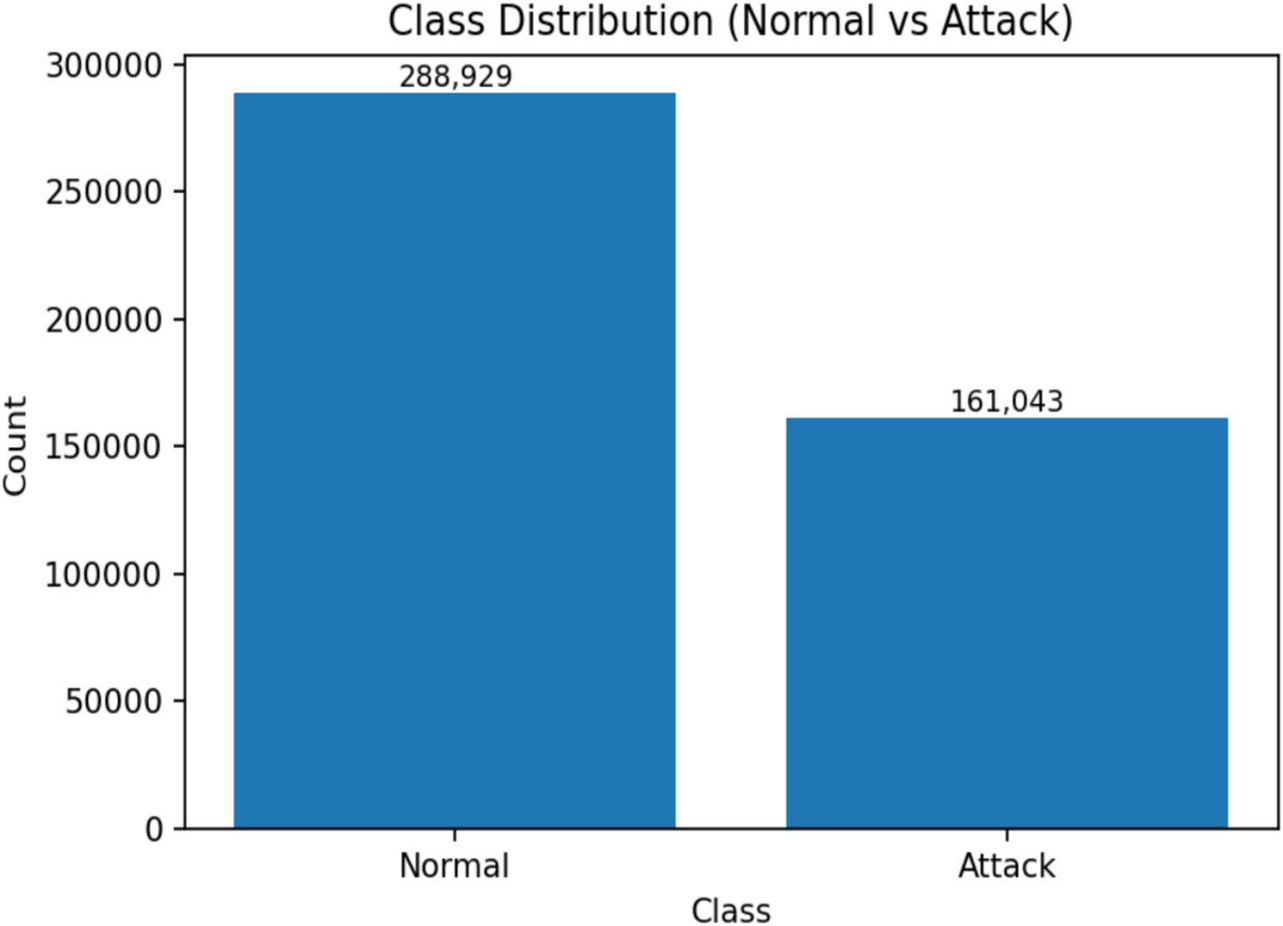
Class distribution of the TON_IoT dataset (Normal vs Attack).

**Fig. 4:**
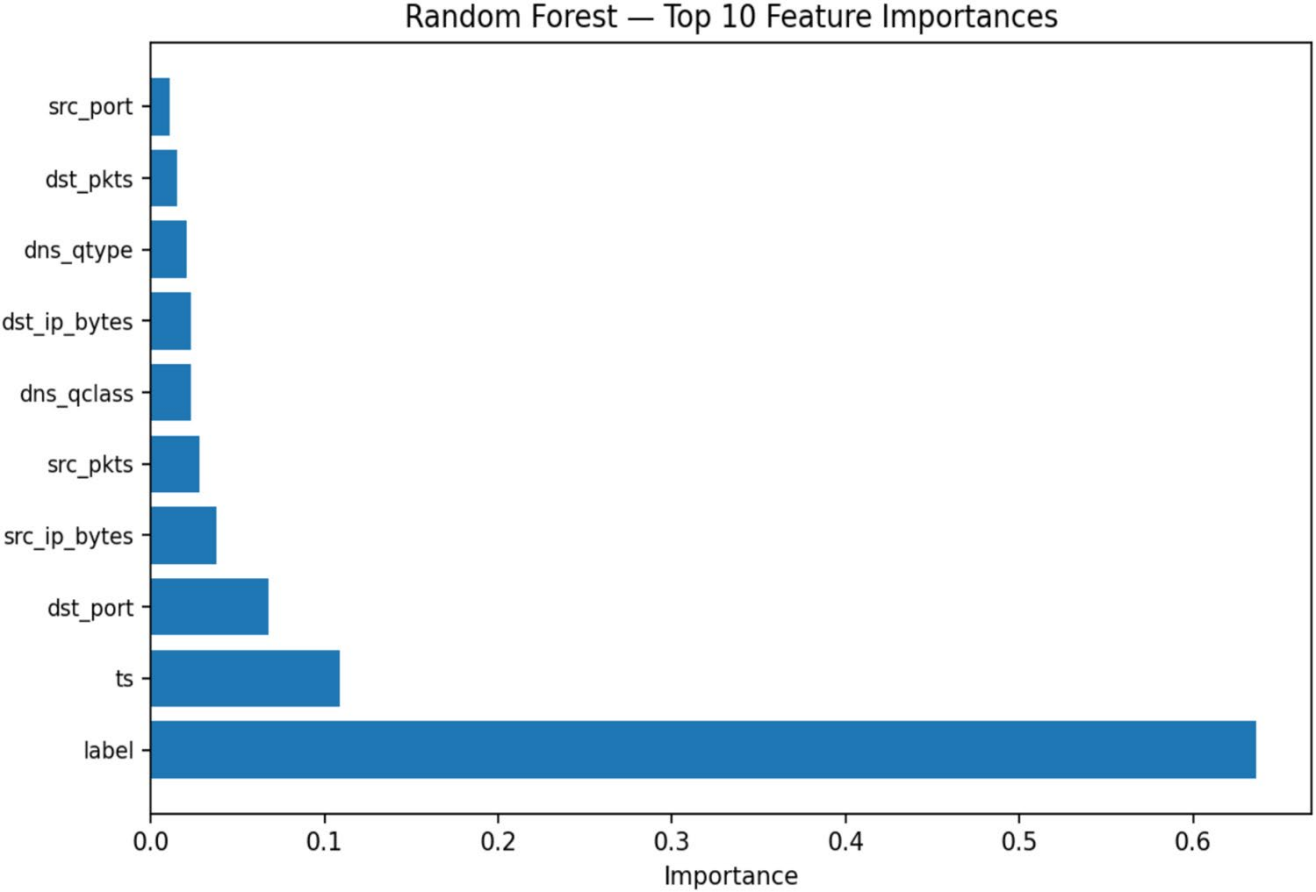
Correlation heatmap of numeric features in the TON_IoT dataset.

**Fig. 5:**
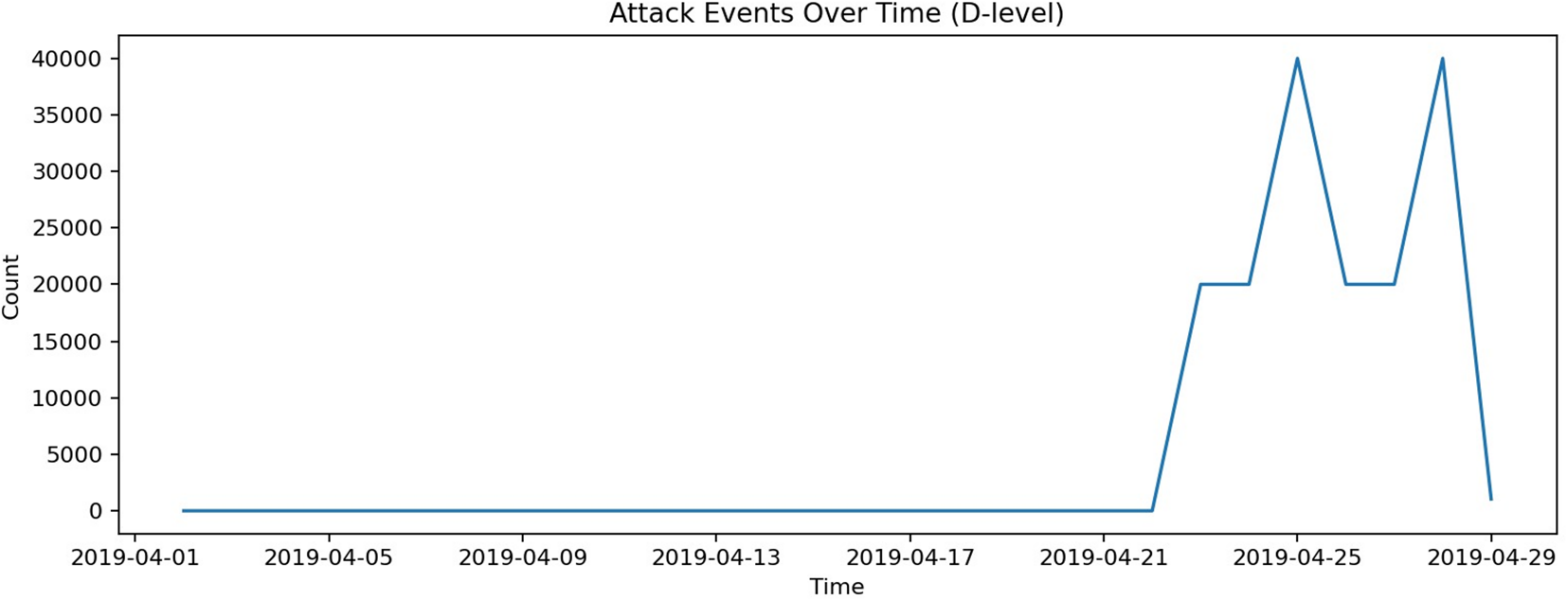
Attack events over time.

This research employs the TON_IoT dataset, developed by the Australian Centre for Cyber Security (ACCS), to design and evaluate the proposed anomaly detection framework. TON_IoT is a widely used benchmark for IoT and IIoTsecurity research. It provides diverse data sources with clearly labeled normal and attack traffic, enabling the development and testing of IDS. The dataset includes both normal events and attack events, with ground-truth labels supporting both binary classification (normal vs. attack) and multi-class classification tasks^27^. The dataset used in our experiments contains 288,929 normal records and 161,043 attack records, ensuring sufficient diversity for anomaly detection tasks. Figure 3 illustrates the distribution of normal and attack samples, while Fig. 4 presents the correlation among numeric features. Attack occurrences over time are also depicted in Fig. 5, showing recurring bursts of malicious activity that highlight the dynamic nature of IoT traffic.

To justify the choice of TON_IoT, we compared it with two widely used alternatives: BoT-IoT and CICIDS2017. As shown in Table 3, unlike BoT-IoT, which is heavily botnet-focused, and CICIDS2017, which is oriented toward traditional IT traffic, TON_IoT offers greater heterogeneity by combining network flows, telemetryand system logs. This makes it particularly well suited for anomaly detection in IoT environments.

**Table 3:** Dataset Comparison.

Dataset	Domain	Data sources	Typical attacks	Strengths	Limitations
TON_IoT	IoT/IIoT	Sensors, OS logs, network	DoS, DDoS, scanning, exfil	Heterogeneous, IoT-specific, labeled	Class imbalance
BoT-IoT	IoT	Network traffic	Botnet-based (DDoS, DoS)	Large scale, detailed attack flows	Highly imbalanced
CICIDS2017	IT	Network traffic	DoS, DDoS, brute force, web	Standard baseline, widely cited	Not IoT-specific

### Dataset preprocessing

Before training and evaluating the models, the dataset was preprocessedthrough three key steps: splitting, filtering and scaling. Using the scikit-learn library, the dataset was divided into a training set (70%) and a testing set (30%).To ensure proper model behavior, the training set was restricted to clean, non-malicious records labeled as normal. This approach is critical for algorithms such as OCSVM and IF, which are unsupervised methods that learn what “normal” traffic looks like. By training exclusively on normal data, these models can more effectively identify deviations during testing, thereby detecting potential anomalies.

### Feature selection

**Fig. 6:**
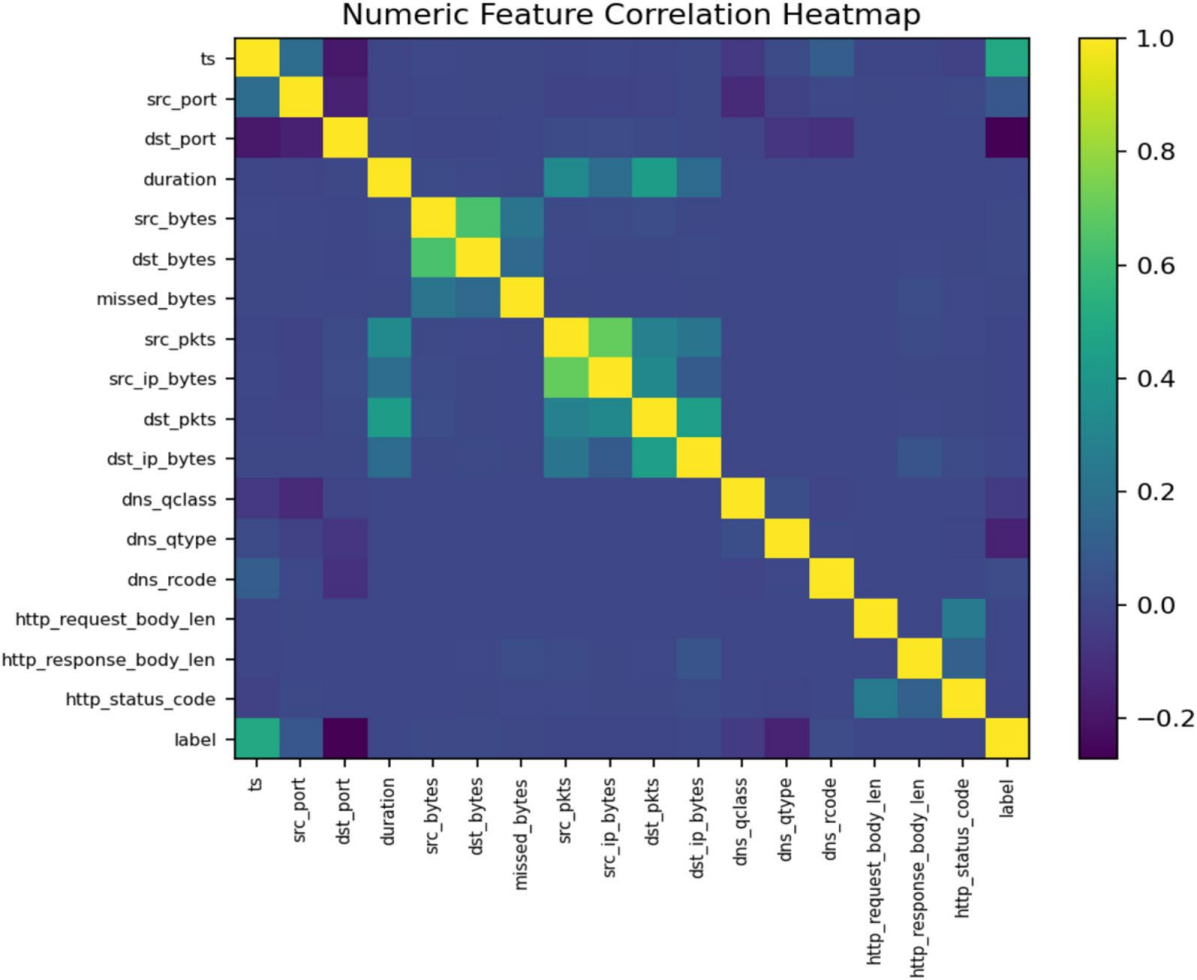
Random Forest–based feature importance ranking.

Choosing the appropriate features is a crucial step in building effective models for anomaly detection. This step also helps reduce the risk of overfitting, accelerates training, and boosts model efficiency^28^. In this research, input X (features)were separated from their correspondingy(labels)during both training and testing. The test dataset labels were transformed into binary format, with ‘1’ denoting anomalies and ‘0’ indicating normal behavior. To determine the most significant features contributing to anomaly detection, we employed a RF classifier, which not only makes predictions but also ranks features by their importance.This approach guarantees that the model is trained on the most relevant attributes, enhancing both efficiency and interpretability.The top-ranked features identified through RF-based analysis are presented in Fig. 6, showing that attributes such aslabel, tsand dst_port contribute significantly to anomaly detection.

### Proposed models

This research employs two unsupervised learning algorithmsi.eOCSVM and IF for anomaly detection on the TON_IoT dataset. A brief overview of each model is provided in the following subsections.

### Isolation Forest (IF)

IF is a widely used unsupervised learning algorithm for detecting anomalies, particularly effective in IoT data scenarios^29^. The method operates by constructing a large ensemble of randomly generated binary trees, known as isolation trees, to isolate individual data points. The underlying concept is based on recursive partitioning—where features are randomly selected and a split value is chosen within the range of that feature’s values. Anomalies, being rare and different from normal patterns, are typically isolated earlier in the partitioning process and thus appear in shorter paths within the trees. Normal instances, on the other hand, require more partitions to isolate and thus end up in deeper branches. This structural distinction allows the model to effectively differentiate outliers from regular data^30^. The performance of IF is evaluated using standard metrics such as accuracy, precision, recall, and F1-score to assess its anomaly detection capability.

### One-Class SupportVector Machine (OCSVM)

OCSVM is a robust anomaly detection technique, widely used for analyzing high-dimensional IoT network traffic.It is particularly useful when only normal data is available during training. OCSVM works by learning a decision boundary that encloses the majority of normal instances. During testing, any data points that fall outside this learned boundary are flagged as anomalies^31^.This method has demonstrated strong performance on benchmark datasets like TON-IOT, effectively distinguishing between benign and malicious network behavior. The diverse set of features available in IoT datasets—such as protocol types, network services, flags, and statistical flow metrics—make them well suited for evaluating OCSVM’s capability in complex IoT environments.

**Algorithm 1:**
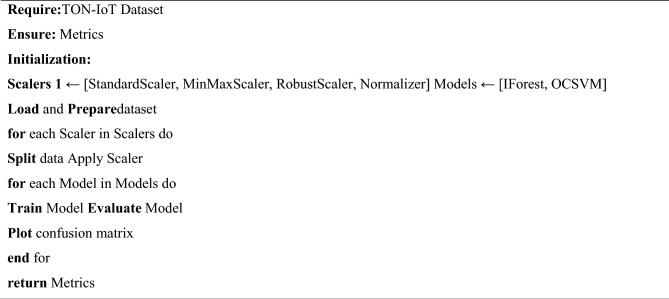
Anomaly detection algorithm.

### Performance evaluation

The performance of the proposed models was evaluated using standard indicators, including accuracy, precision, recall, F1-score and the confusion matrix^32^. These indicators are derived from four fundamental parameters:

- Truepositive(TP):Caseswhereanticipatedandactualoutcomesarepositive.
- Truenegative(TN):Caseswhereanticipatedandactualoutcomesarenegative.
- Falsepositive(FP):Caseswhere real outcomeisnegativebutthemodelanticipatedittobepositive.
- Falsenegative(FN):Caseswhereactualvalueispositivebutthemodelanticipatedthatitisnegative.

### Hyper parameter configuration of models

To ensure optimal performance and reproducibility, hyperparameters for the OCSVMmodel were fine-tuned using a Grid Search approach combined with a custom scoring function based on the F1-score. Since OCSVM model is trained only on normal data, we used a subset of 500 normal samples from the training set to reduce computational cost during hyperparameter search. The search space included kernel = [‘rbf’], nu = [0.1, 0.5], and gamma = [‘scale’]. The best-performing model had kernel=‘rbf’, nu=0.1, and gamma=‘scale’. For the Isolation Forest (IF) model, we used commonly accepted defaults with n_estimators=100 and contamination=‘auto’. These configurations were selected based on performance metrics obtained from the validation set and are reported to ensure reproducibility.The hyperparameter configurations used for OCSVM and IF are summarized in Table 4.

**Table 4:** Hyper parameter configuration of models.

Model	Tuning method	Hyper parameters considered	Best parameters selected
One-Class SVM	Grid Search (threefold CV on normal subset)	kernel: [‘rbf’] gamma: [‘scale’] nu: [0.1, 0.5]	kernel = ‘rbf’, gamma = ‘scale’, nu = 0.1
Isolation Forest	Manual	n_estimators: [100] contamination: ‘auto’	n_estimators = 100, contamination = ‘auto’

### Ensemble-based anomaly detection with CSAD

In this research we propose a lightweight fusion-based approach named (CSAD) that combines anomaly scores from OCSVM and IF. The combined score is calculated using average/max strategy, followed by a thresholding step to flag anomalies. This approach leverages the strengths of both models and introduces a lightweight ensemble without complex overhead.The performance evaluation of these models is presented in the next section, where we report experimental results on the TON_IoT dataset.

### Implementation and result analysis

The following sections discuss the findings of the assessment of our model. Two algorithms IF and OCSVM are trained, tested, evaluatedand compared using the TON_IoT dataset. Several performance metrics used for accessing the results as discussed in Section III-E. The proposed model successfully classified the data into normal or attack categories.

### Model performance on TON_IoT dataset

**Fig. 7:**
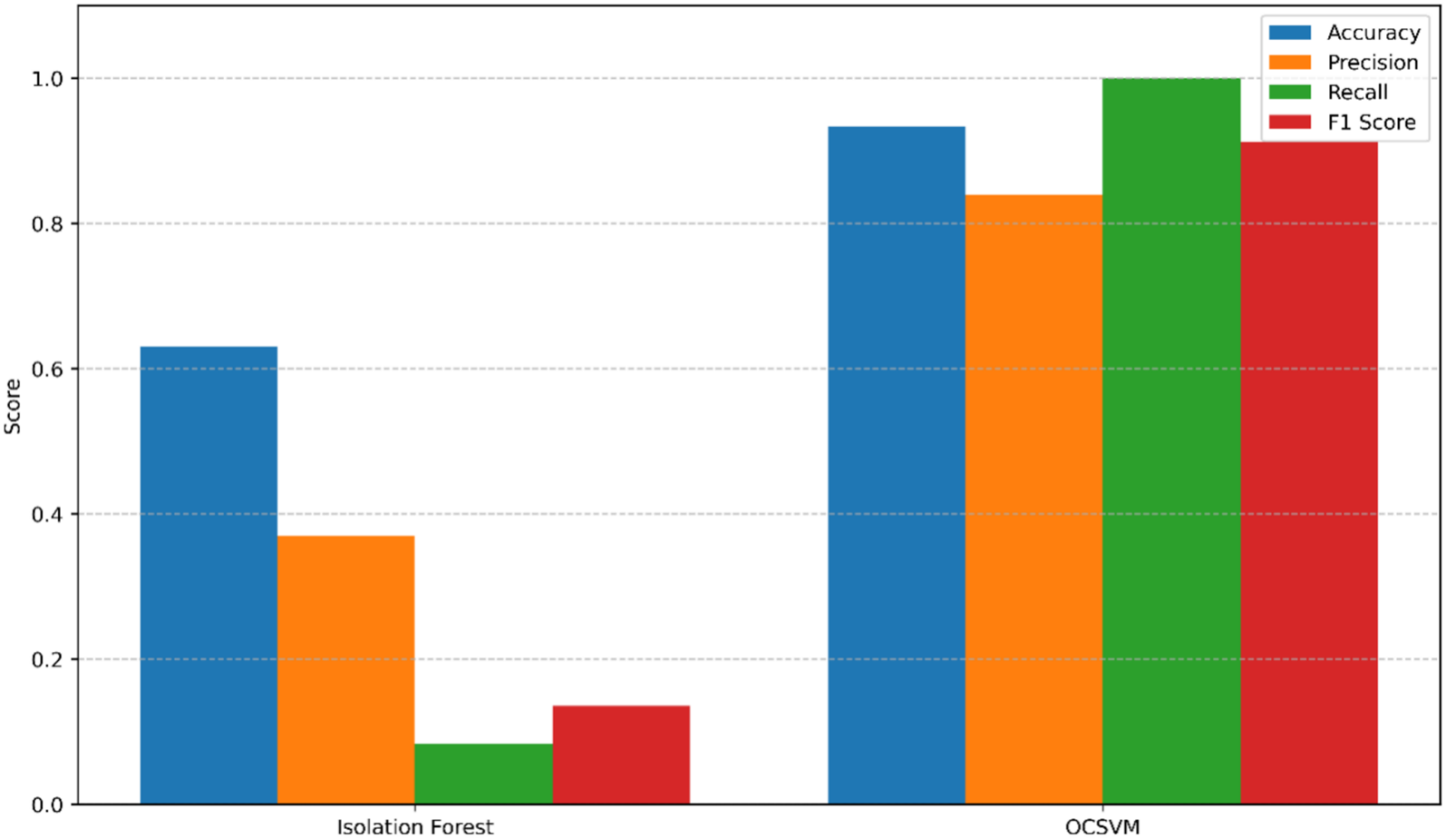
Performance metricscomparison ofIF and OCSVM.

**Fig. 8:**
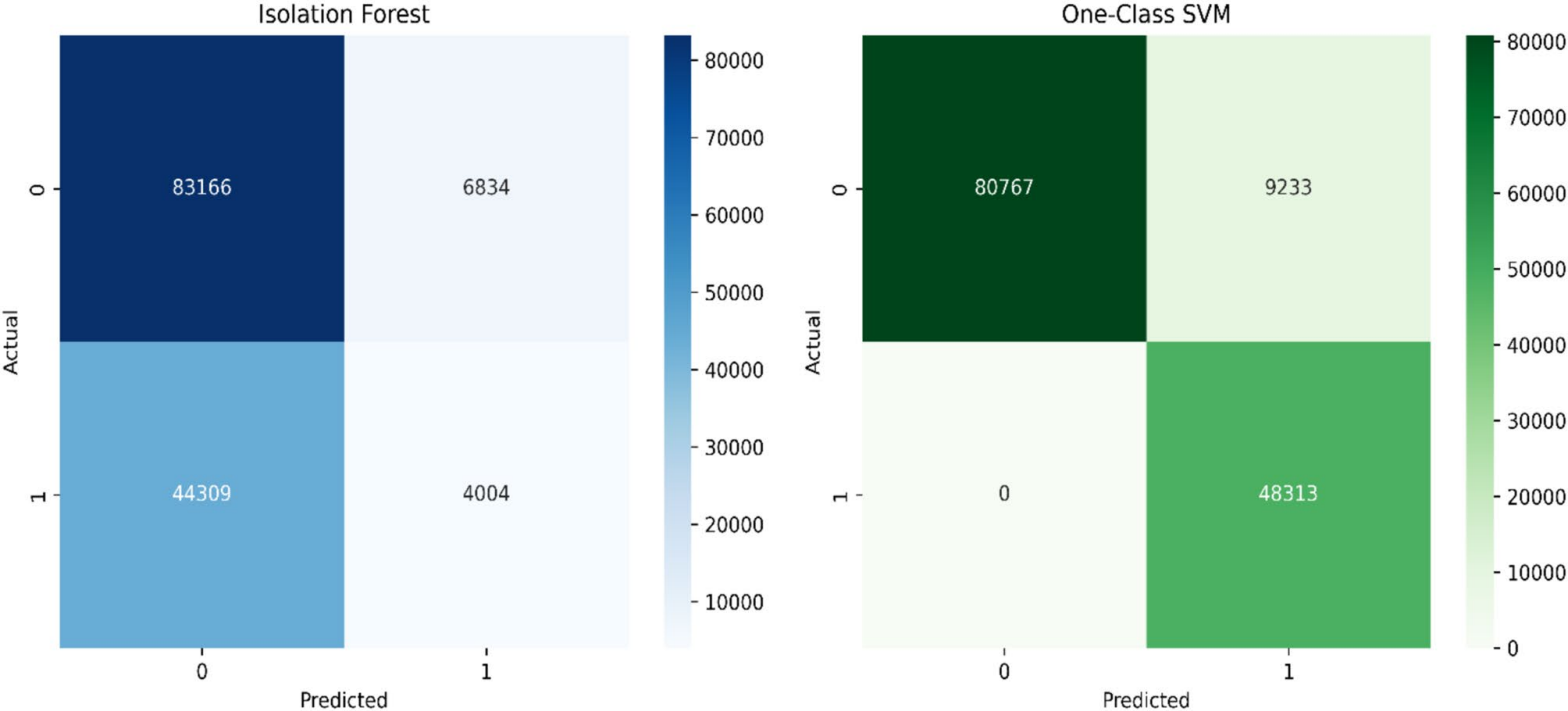
Confusion matrix of OCSVM and IF.

We evaluated the performance of IF and OCSVMon the TON_IoT dataset using binary classification (normal vs. attack). The graphical representation of the model performance is displayed in Fig. 7 where the x-axis represents the anomaly detection algorithms and the y-axis indicates the corresponding metrics percentages. The confusion matrices for both IF and OCSVM are illustrated in Fig. 8, highlighting the true positives (TP), true negatives (TN), false positives (FP), and false negatives (FN) identified by each model. The results indicate that OCSVM consistently outperforms IF under normal conditions.

### Comparative evaluation with CSAD

**Fig. 9:**
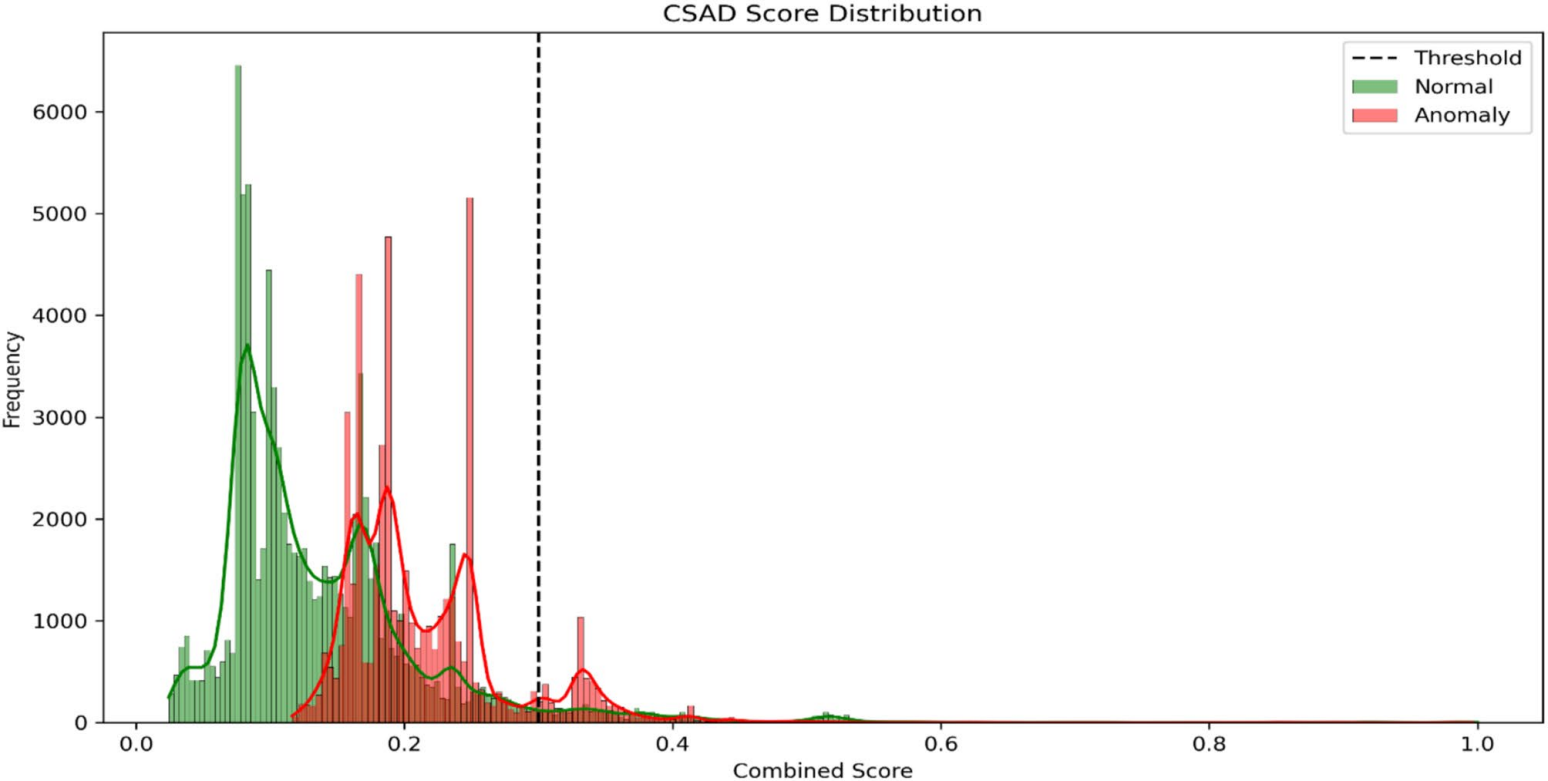
Score distribution across models.

In addition to the individual performance of OCSVM and IF, we propose a lightweight fusion-based approach named Combined Scoring Anomaly Detection (CSAD). CSAD integrates the anomaly scores of OCSVM and IF to improve overall detection performance through score-level fusion. We evaluated CSAD using a threshold-based ensemble, where the average anomaly scores were used to predict outliers. To enhance sensitivity, the decision threshold was fine-tuned to 0.3 and feature scaling was applied via StandardScaler. Although OCSVM achieved the highest anomaly detection performance individually (with precision of 0.84 and recall of 1.00), CSAD outperformed IF significantly (raising precision from 0.37 to 0.55 and recall from 0.08 to 0.12), confirming that score fusion improves robustness and consistency. These results show that CSAD provides a competitive balance between high recall and reduced false positives in complex IoT scenarios. Table 5 summarizes the performance comparison among the baseline models (OCSVM and IF) and the proposed CSAD ensemble,while Fig. 9 visualizes the score distributions across models.

**Table 5:** Performance comparison of OCSVM, IF and CSAD on TON_IoT test set.

Model	Accuracy	Precision (Anomaly)	Recall (Anomaly)	F1 Score (Anomaly)
OCSVM	93.32%	0.84	1.00	0.91
Isolation Forest	63.02%	0.37	0.08	0.13
CSAD (Proposed)	66.00%	0.55	0.12	0.20

### Comparative analysis with State-of-the-Art methods

To contextualize our results,we compare the proposed approach with recent deep learning models evaluated on the TON_IoT dataset^33^. While deep learning has demonstrated promise in various IoT security applications^34^, its practical implementation in resource-constrained IoT environments often faces challenges due to high computational overhead and large data requirements. In contrast, our research prioritizes lightweight algorithms like IF and OCSVM^35^with their ensemble via CSAD, to achieve a balance between detection accuracy and deployment feasibility.Table 6 provides a comparative overview of the performance of our models against several recent deep learning-based IDSs on the TON_IoT dataset.

**Table 6:** Comparative analysis with State-of-the-Art methods.

References	Year	Model	Accuracy	F1 Score
Our paper	2025	OCSVM	93.32%	0.91
Our paper	2025	IF	63.02%	0.13
Our paper	2025	CSAD	66.00%	0.20
Ahmad et al.^35^	2021	CNN	99.00%	-
Aktar et al.^2^	2024	Deep SVDD + Contractive AE	99.57%	99.25%
Ayad et al.^5^	2025	OC-ASAE + DNN	99.99%	97.69%

### Feature importance analysis

**Fig. 10:**
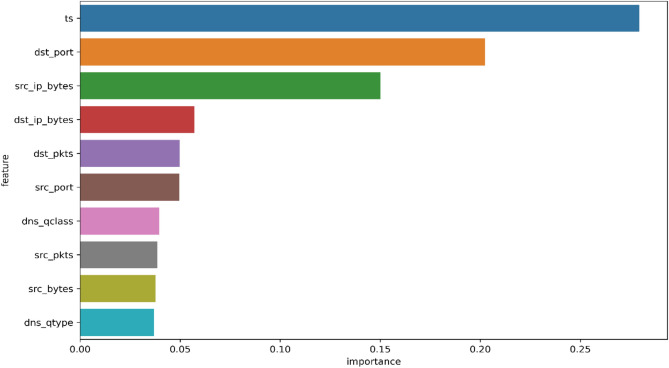
Top 10 most important features identified by the RF model on the TON_IoT dataset.

To support the feature selection process, we performed a feature importance analysis using a RF classifier. This analysis identifies which features in the TON_IoT dataset most strongly influence the model’s predictions. Figure 10 presents the top 10 most important features ranked by their contribution to the model. The ts (timestamp) feature was found to be the most influential, followed by dst_port, src_ip_bytes, and dst_ip_bytes. These features are closely tied to network traffic behavior and are critical for distinguishing between benign and anomalous activity. The results of this analysis not only confirm the relevance of the selected features but also provide insights into which attributes play the most significant role in the anomaly detection process.

### Model interpretability with LIME

**Fig. 11:**
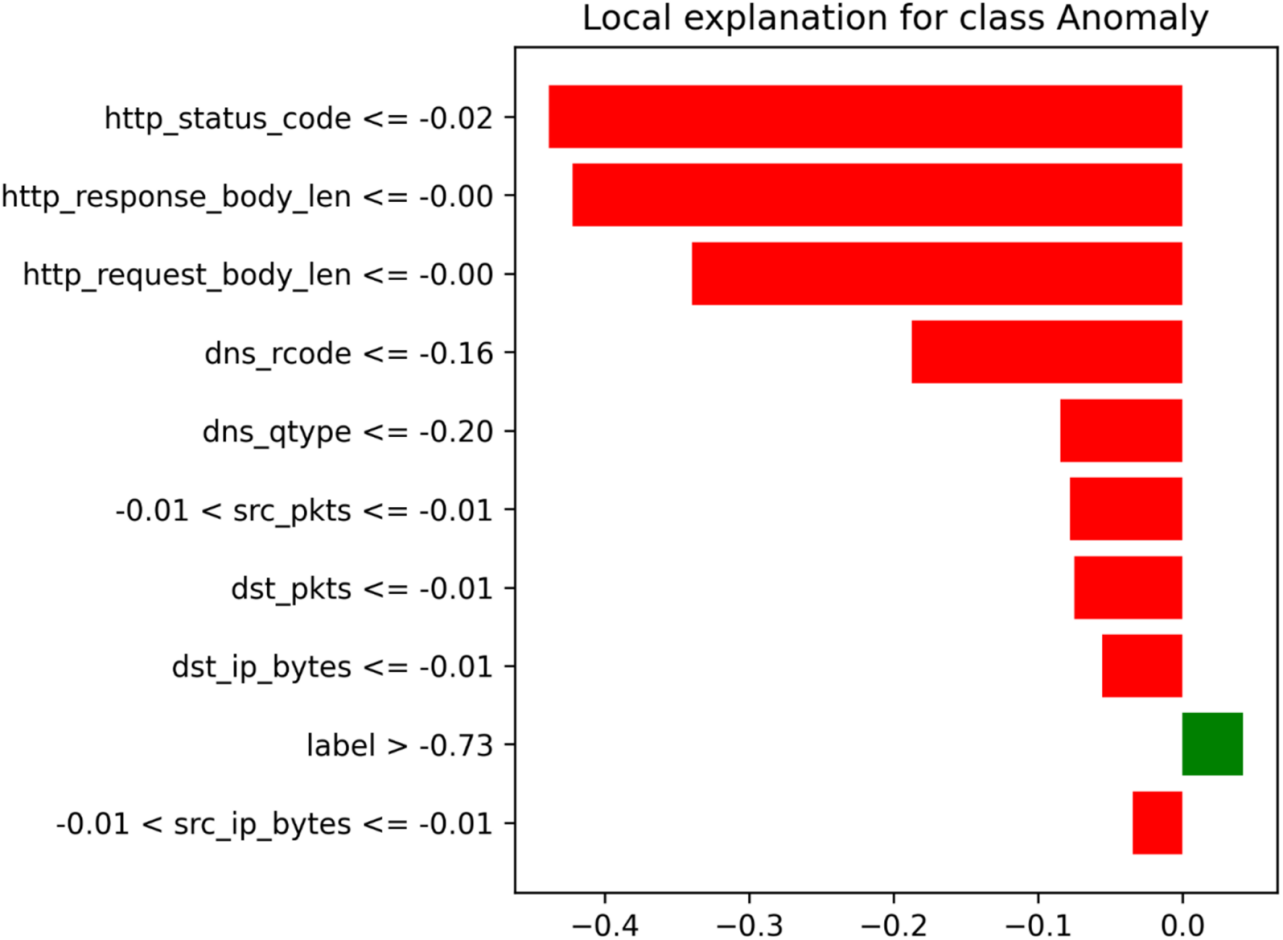
LIME-based local explanation for an anomalous instance predicted by the OCSVM model.

To improve interpretability and address the black-box nature of model like OCSVM, we used the Local Interpretable Model-Agnostic Explanations (LIME) framework. LIMEgenerates localized, feature-level explanations for anomalous predictions making the model’s decision process more transparent.As illustrated in Fig. 11, we visualized the top 10 features that contribute to an instance being classified as an anomaly by the OCSVM model.Key attributes such as http_status_code, http_response_body_len, and dns_rcode were identified as major contributors to the anomaly score.Integrating the LIME framework enhances the model’s reliability and practical value in real-world IoT intrusion detection applications.

### Statistical validation and cross-validation performance

**Fig. 12:**
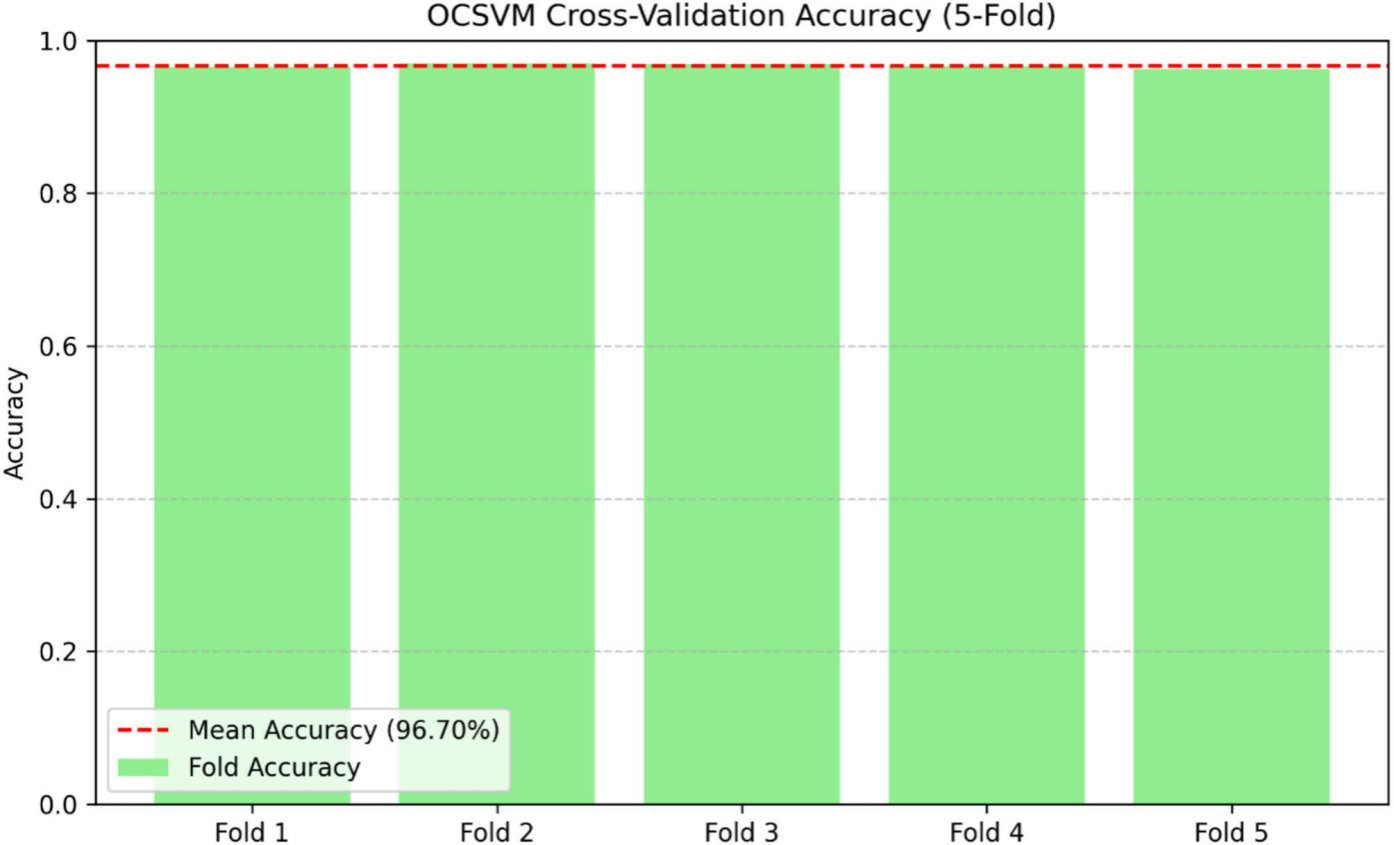
OCSVM fivefold cross-validation accuracy.

**Fig. 13:**
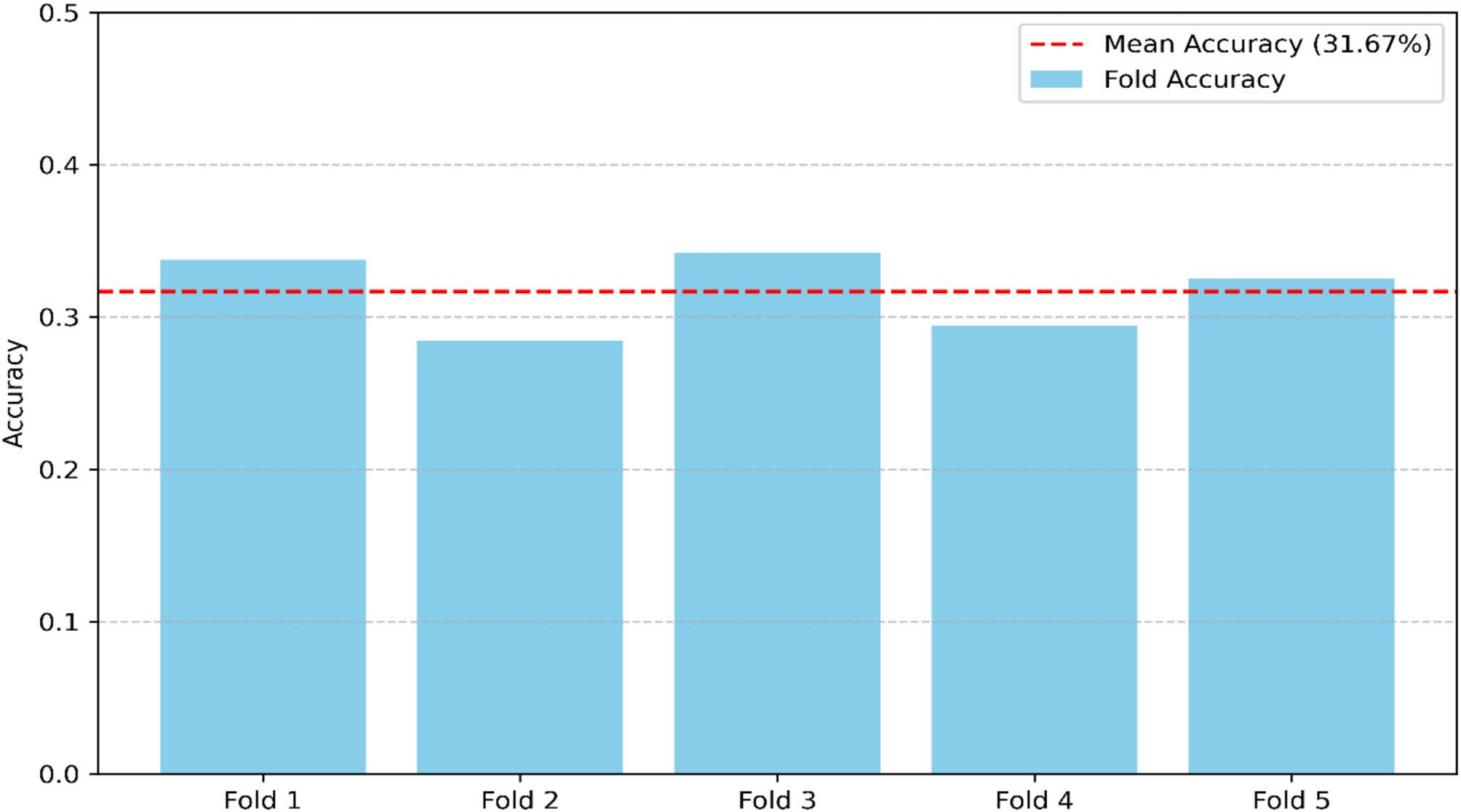
IF fivefold cross-validation accuracy.

To ensure the robustness and generalizability of the anomaly detection models, we conducted fivefold cross-validation for both OCSVM and IF. In this approach, the dataset is divided into five equal partitions. The model is iteratively trained on four partitions and tested on the remaining one and the process is repeated until each partition has served as a test set once.The mean accuracy across all folds was reported to minimize potential bias from a single train-test split. For OCSVM, a high mean accuracy of 96.70% with minimal variance across folds confirmed stable performance. In contrast, IF showed a lower mean accuracy of 31.67%, indicating weaker anomaly detection capability. These results are visualized in Figs. 12 and 13. This cross-validation approach strengthens the statistical validity of the findings and reduces the risk of overfitting, providing stronger evidence for the reliability of the proposed framework.

### Robustness evaluation

**Fig. 14:**
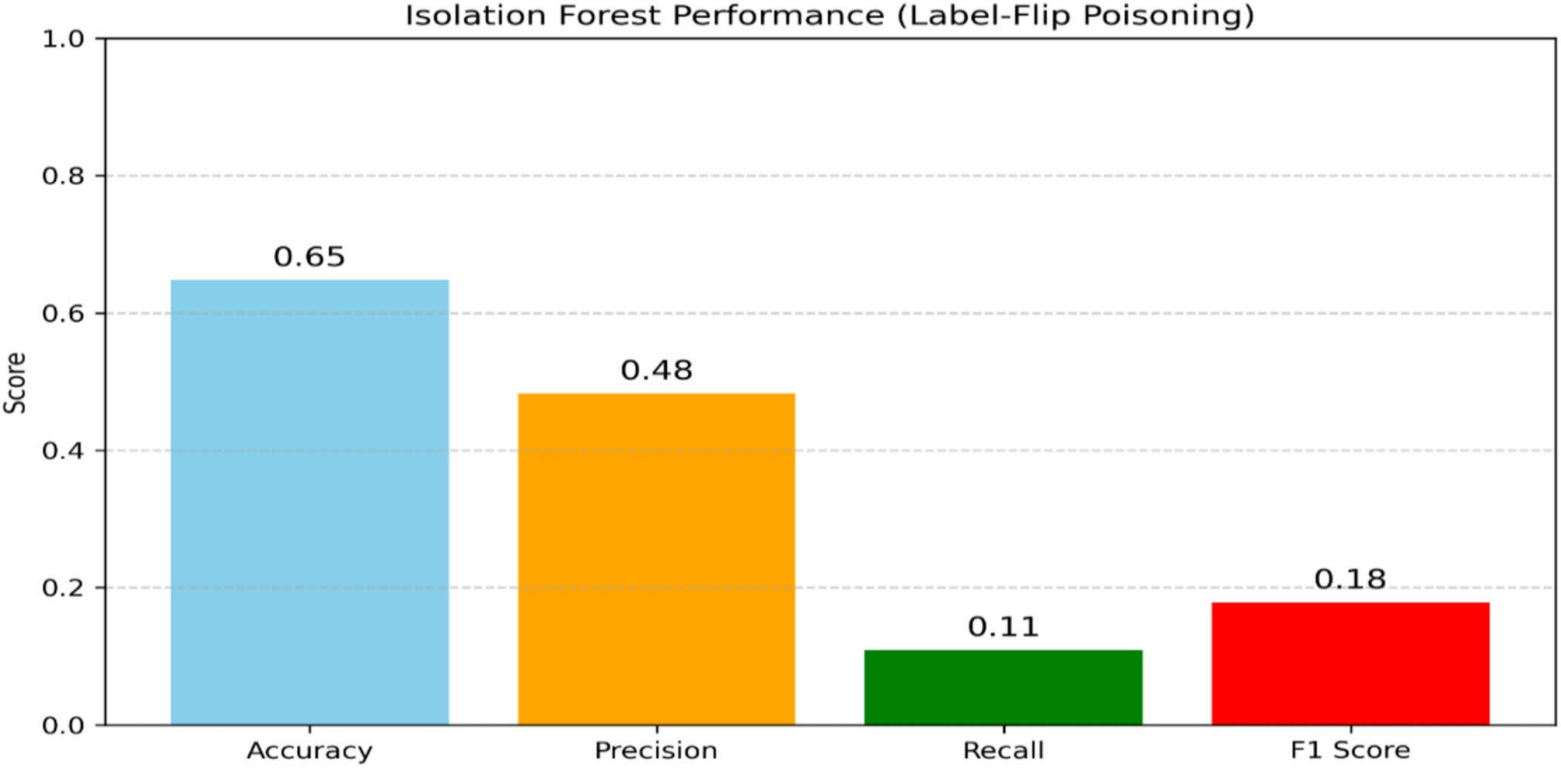
IF performance with Label-Filp Positioning.

**Fig. 15:**
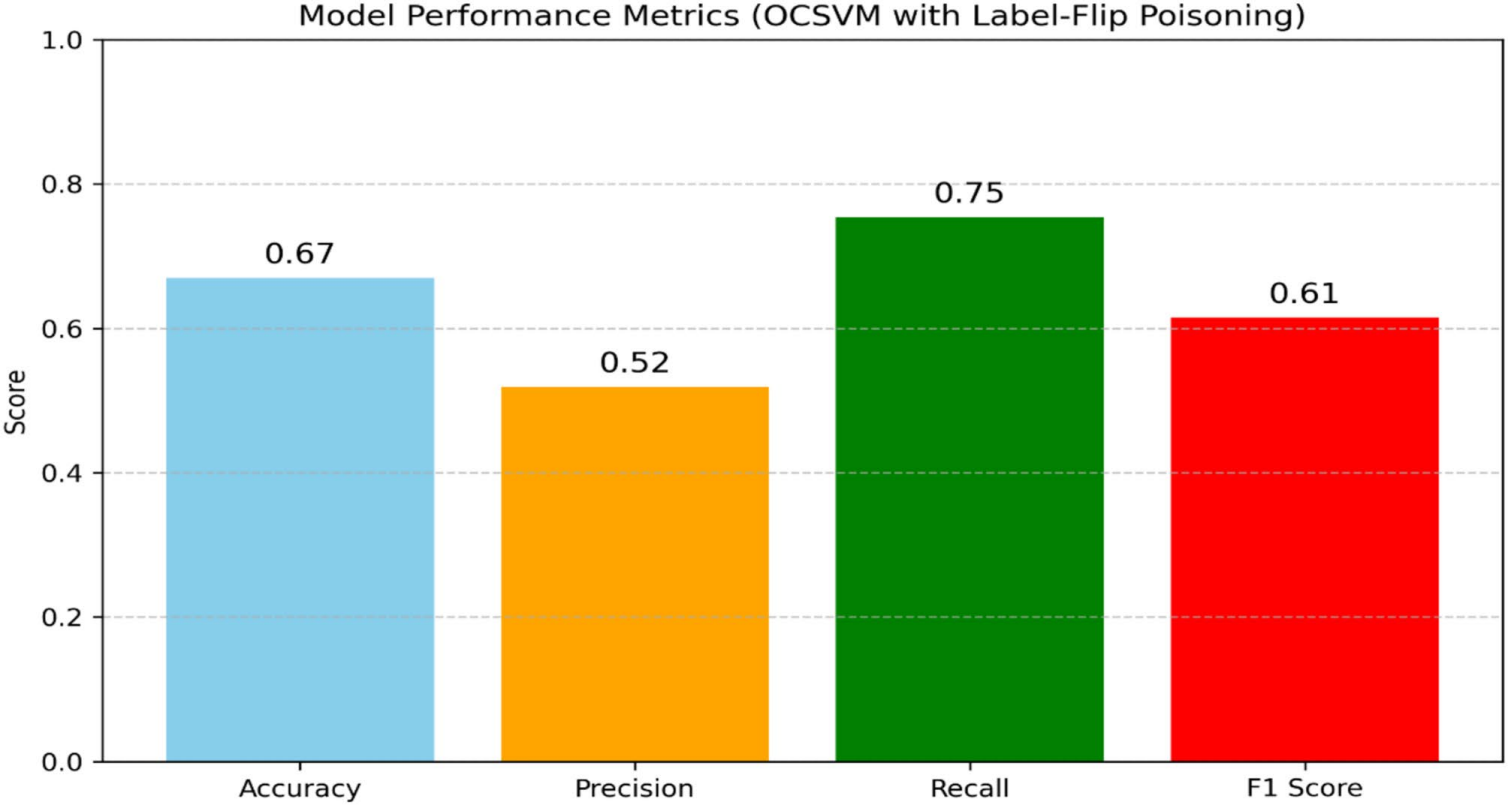
OCSVM performance with Label-Filp Positioning.

In the context of anomaly detection for IoT networks, adversarial attacks such as evasion attacks (e.g., Fast Gradient Sign Method, FGSM) and poisoning attacks (e.g., label-flip) are highly relevant. To assess the robustness of our proposed models against adversarial poisoning we implemented and evaluated a label-flip poisoning attack, a common strategy in which a portion of attack samples in the training set are mislabeled as normal. Specifically, we flipped 30% of attack-labeled samples to appear as normal, simulating a poisoning scenario where malicious inputs are intentionally mislabeled to deceive the learning algorithm. We then retrained our models (IF or OCSVM) on the poisoned dataset and comparedtheir performance against the clean models. The results demonstrate that the inclusion of mislabeled attack samples in the training data significantly reduces model’s performance, underscoring the importanceof robustness against poisoning attacks. Figures14 and 15 present bar charts comparing the performance of OCSVM and IF before and after thelabel-flip poisoning attack.

**Fig. 16:**
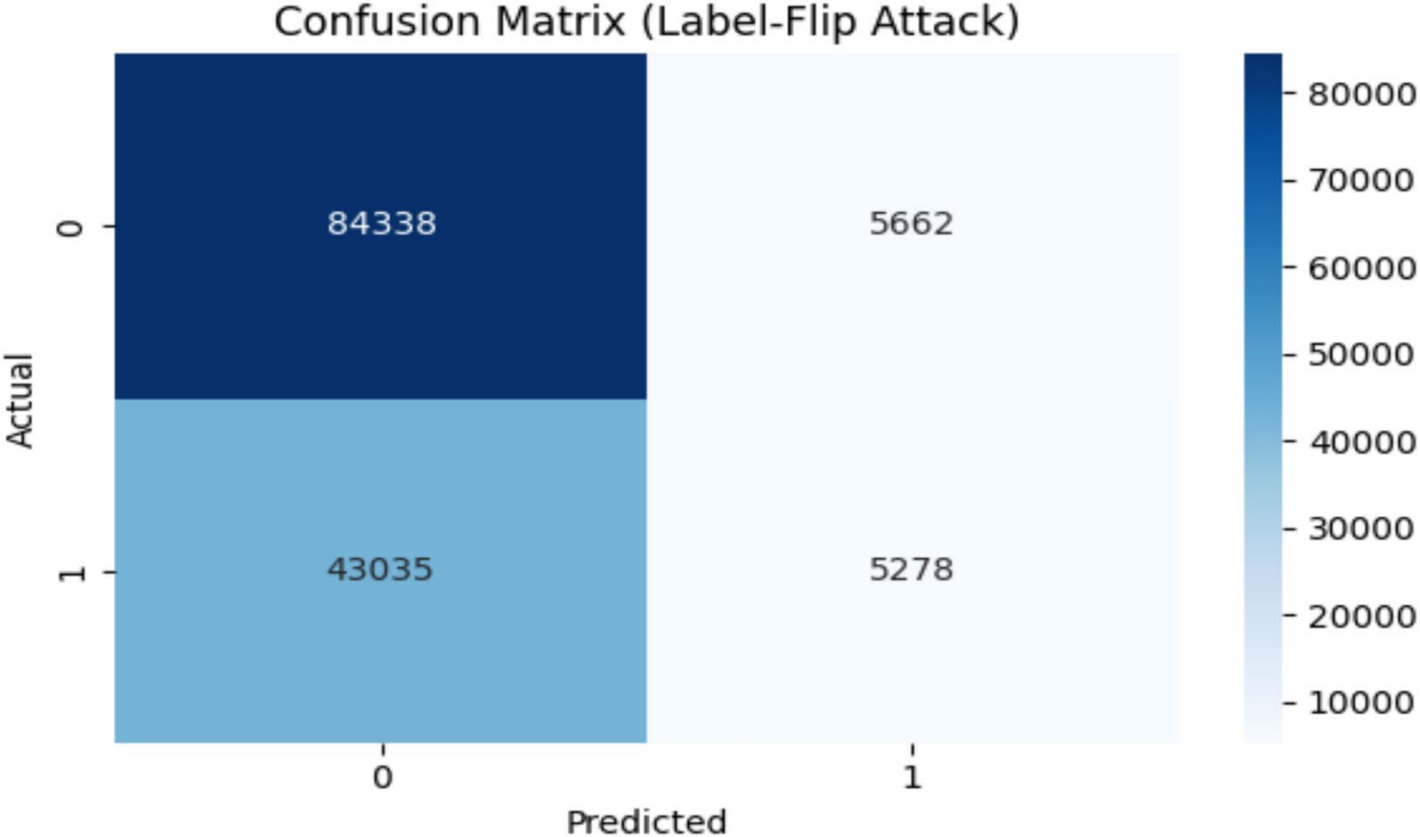
IF confusion matrix after Label-Flip Positioning.

**Fig. 17:**
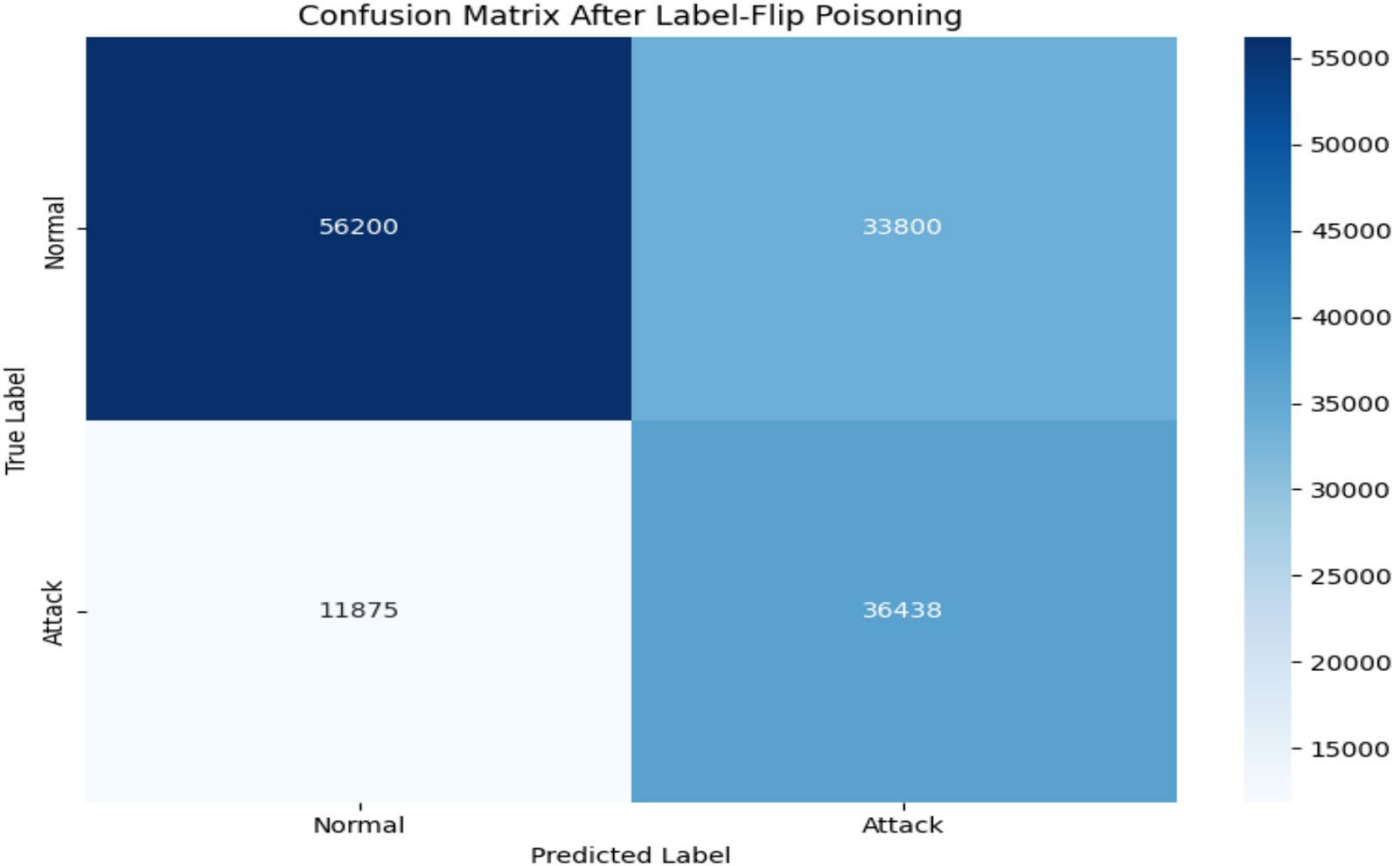
OCSVM confusion matrix after Label-Flip Positioning.

Figure 16 shows confusion matrix of IF algorithm and Figure 17 shows confusion matrix of OCSVM algorithm after applying a label-flip poisoning attack.

Beyond numerical performance, it is important to interpret the significance of these findings and situate them in the broader context of IoT security research. This is discussed in the next section.

## Discussion

The findings of this study provide important insights into the use of unsupervised learning techniques for anomaly detection in IoT networks. Among the evaluated models, the OCSVM consistently outperformed both IF and the ensemble methodCSAD. This superior performance can be attributed to OCSVM’s ability to define flexible decision boundaries in high-dimensional data, making it particularly effective for capturing subtle deviations in complex IoT traffic. In contrast, IF, which relies on random partitioning, was less effective when anomalies closely resembled normal traffic patterns. The comparative analysis also showed that CSAD offered moderate improvements in recall and reduced false positives compared to IF alone, yet it did not surpass OCSVM. This indicates that while ensemble methods can stabilize performance, their benefit is limited if the base models do not provide complementary strengths.

A further contribution of this study is the integration of interpretability and robustness assessment, aspects often neglected in IoT intrusion detection research. By applying RF–based feature importance and LIME explanations, the framework provided transparency in decision-making, highlighting features such as port usage and flow statistics as critical indicators of anomalous behavior. This addresses the common concern that ML–based IDSs function as “black-box” models, thereby enhancing trust and practical applicability. Additionally, the robustness experiments under label-flip poisoning attacks demonstrated a measurable decline in model performance, underscoring the vulnerability of traditional unsupervised approaches to adversarial manipulation. This result emphasizes the importance of considering adversarial resilience in the design of next-generation IDS solutions.

In the broader context of related work, our results confirm that lightweight unsupervised models can be viable alternatives to computationally expensive deep learning solutions, particularly in resource-constrained IoT deployments. While deep learning approaches often report higher accuracy on benchmark datasets, their practical applicability in IoT environments is limited by high training costs, large labeled data requirements and deployment complexity. Our findings demonstrate that unsupervised methods like OCSVM, when combined with robust feature analysis and interpretability tools, can deliver competitive detection performance with minimal overhead.

At the same time, some limitations remain. Our current framework was restricted to binary classification (normal vs. attack), preventing detailed differentiation among specific attack categories. Furthermore, the experiments were conducted in an offline setting, without testing real-time scalability across large and heterogeneous IoT systems. These constraints highlight avenues for future research, including extending anomaly detection to multi-class attack scenarios, integrating explainable AI methods such as SHAP and assessing performance under more sophisticated adversarial conditions such as evasion attacks. Finally, the study is concluded by summarizing the key insights, acknowledging limitations and outlining directions for future research.

### Conclusion and future work

Cybersecurity has become a critical challenge due to the rapid expansion of IoT devices. Their interconnected and resource-constrained nature makes them vulnerable to diverse security and privacy threats. In this study, we evaluated two unsupervised machine learning models—IF and OCSVM—on the TON_IoT dataset to detect anomalies in IoT network traffic. A comparative analysis also introduced a lightweight fusion approach,CSAD, which integrates anomaly scores from both models. Experimental results revealed that OCSVM consistently outperformed IF and CSAD in terms of accuracy, precision, and recall, providing a reliable solution for IoT anomaly detection. These findings highlight that even traditional unsupervised techniques, when applied systematically, can provide effective detection in resource-limited environments. In addition, this work contributes by applyingLIME to improve interpretability, assessing model robustness under label-flip poisoning attacks and employing RF-based feature importance, fivefold cross-validation and hyperparameter tuning to ensure statistical rigor and reproducibility.

Despite these promising results, several limitations remain. The current framework is restricted to binary classification, limiting its ability to distinguish among different categories of cyber-attacks. Moreover, the models were not tested for real-time deployment or scalability across large and heterogeneous IoT networks. Issues such asadversarial robustness (e.g., resistance to poisoning attacks) and the integration of interpretability techniques are also open challenges.

Future work will focus on addressing these limitations. Specifically, we aim to extend the framework toward multi-class classification to differentiate between various IoT attack types and to evaluate its performance on larger and combined datasets that better represent real-world IoT traffic. In addition, we plan to incorporate explainable AI tools such as SHAP to improve interpretability and support security analysts in decision-making. Exploring adversarial resilience under poisoning or evasion attacks and assessing the scalability of the models in real-time IoT environments will also be prioritized. Furthermore, hybrid and ensemble approaches that combine deep learning with lightweight models may provide a pathway to balance detection accuracy with efficiency, enabling practical adoption in real-world IoT systems.

## Data Availability

The datasets used and/or analyzed during the current study are available from the corresponding author, Muhammad Zeeshan Babar* (Email: m.babar@hw.ac.uk) and Dr. Waseem Abbasi (email: waseemabbasi97@gmail.com), upon reasonable request.
